# Assessment of mitral bioprostheses using cardiovascular magnetic resonance

**DOI:** 10.1186/1532-429X-12-36

**Published:** 2010-06-23

**Authors:** Florian von Knobelsdorff-Brenkenhoff, André Rudolph, Ralf Wassmuth, Jeanette Schulz-Menger

**Affiliations:** 1 Working Group on Cardiovascular Magnetic Resonance, Medical University Berlin, Charité Campus Buch, HELIOS Klinikum Berlin-Buch, Department of Cardiology and Nephrology, Schwanebecker Chaussee 50, 13125 Berlin, Germany

## Abstract

**Background:**

The orifice area of mitral bioprostheses provides important information regarding their hemodynamic performance. It is usually calculated by transthoracic echocardiography (TTE), however, accurate and reproducible determination may be challenging. Cardiovascular magnetic resonance (CMR) has been proven as an accurate alternative for assessing aortic bioprostheses. However, whether CMR can be similarly applied for bioprostheses in the mitral position, particularly in the presence of frequently coincident arrhythmias, is unclear. The aim of the study is to test the feasibility of CMR to evaluate the orifice area of mitral bioprostheses.

**Methods:**

CMR planimetry was performed in 18 consecutive patients with mitral bioprostheses (n = 13 Hancock^®^, n = 4 Labcore^®^, n = 1 Perimount^®^; mean time since implantation 4.5 ± 3.9 years) in an imaging plane perpendicular to the transprosthetic flow using steady-state free-precession cine imaging under breath-hold conditions on a 1.5T MR system. CMR results were compared with pressure half-time derived orifice areas obtained by TTE.

**Results:**

Six subjects were in sinus rhythm, 11 in atrial fibrillation, and 1 exhibited frequent ventricular extrasystoles. CMR image quality was rated as good in 10, moderate in 6, and significantly impaired in 2 subjects. In one prosthetic type (Perimount^®^), strong stent artifacts occurred. Orifice areas by CMR (mean 2.1 ± 0.3 cm^2^) and TTE (mean 2.1 ± 0.3 cm^2^) correlated significantly (r = 0.94; p < 0.001). Bland-Altman analysis showed a 95% confidence interval from -0.16 to 0.28 cm^2 ^(mean difference 0.06 ± 0.11 cm^2^; range -0.1 to 0.3 cm^2^). Intra- and inter-observer variabilities of CMR planimetry were 4.5 ± 2.9% and 7.9 ± 5.2%.

**Conclusions:**

The assessment of mitral bioprostheses using CMR is feasible even in those with arrhythmias, providing orifice areas with close agreement to echocardiography and low observer dependency. Larger samples with a greater variety of prosthetic types and more cases of prosthetic dysfunction are required to confirm these preliminary results.

## Background

The hemodynamic evaluation of heart valve bioprostheses is based on a comprehensive assessment of transprosthetic pressure gradients and orifice area, preferably by using transthoracic echocardiography (TTE) [1]. However, TTE is frequently limited due to restricted acoustic windows following cardiac surgery and due to methodical concerns [2]. In particular, the assessment of the prosthetic orifice area, which is the main parameter used to differentiate between a normal functioning prosthesis, prosthetic stenosis and patient-prosthesis mismatch, often remains a challenge.

Recently, cardiovascular magnetic resonance (CMR) has been identified as an accurate non-invasive tool for the assessment of the orifice area of bioprostheses implanted in the aortic position. The orifice areas obtained by CMR planimetry agreed well with those obtained by TTE and transesophageal echocardiography, and artifacts caused by flow turbulences and prosthetic material were rare [3].

Based on these experiences with aortic bioprostheses, and on the established application of CMR to assess the severity of native mitral stenosis [4,5], it would be desirable to apply this method to bioprostheses implanted in the mitral position. However, whether CMR can be applied to mitral bioprostheses is unclear, particularly due to the frequent coincidence of arrhythmias. Therefore, the aim of this study was to determine the feasibility of CMR to quantify the orifice area of mitral bioprostheses and compare the results to TTE.

## Methods

### Patient population

The inclusion criterion for entry into the study was the presence of a biological mitral prosthesis; exclusion criteria were general contraindications for CMR [6]. Screening the database of all patients who underwent echocardiography at our hospital between 2007 and 2009, we identified 138 patients who had undergone mitral valve replacement. Eighteen patients were eligible for the study. Their characteristics and distribution of valve types are depicted in Table 1. One hundred and twenty patients did not meet the inclusion criteria, since (possible nomination of ≥1 reason in each subject) the mitral prosthesis was a mechanical device (n = 74), due to the presence of a pacemaker (n = 29) or internal converter defibrillator (n = 8), because they refused to participate (n = 13) or had claustrophobia (n = 3). The study was carried out in compliance with the Declaration of Helsinki, the institutional ethics committee approved the study (EA3/015/07), and all patients gave written informed consent.

**Table 1:** Patient characteristics and distribution of prosthetic valve types

Number of patients completing TTE and CMR	18
Gender (male/female)	7/11
Mean age (range) at study enrollment [years]	73.7 ± 5.7 (64.0 - 82.8)
Mean time (range) since mitral valve replacement [years]	4.9 ± 3.5 (0.6 - 10.9)
Mean left ventricular ejection fraction (range) [%]	44.6 ± 15.3 (10 - 64)
Mean left atrial area [cm^2^]	31.1 ± 11.1 (18-58)
Native mitral valvular lesion	
Mitral regurgitation	11
Mitral stenosis	4
Combined mitral lesion	3
Heart rhythm	
Sinus rhythm	6
Atrial fibrillation	11
Frequent ventricular extrasystoles	1
Prosthetic types	
Medtronic Hancock^®^	13
Labcore^®^	4
Carpentier Edwards Perimount^®^	1
Prosthetic sizes	
27	4
29	7
31	6
33	1

### Transthoracic echocardiography

TTE was performed on state-of-the-art ultrasound systems (General Electric Healthcare, Vivid 7, Waukesha, USA; Philips Medical Systems, IE33, Andover, USA) in accordance with international guidelines [1,7]. In the presence of arrhythmia, each measurement was repeated at least five times and a mean result was calculated. Peak and mean transprosthetic pressure gradients were obtained using the modified Bernoulli equation. Prosthetic orifice area was calculated based on the pressure half time [8].

### Cardiovascular magnetic resonance

All patients underwent CMR in a clinical 1.5-Tesla MR scanner (Magnetom Avanto, Siemens Healthcare, Erlangen, Germany). For prosthetic orifice visualization, steady-state free-precession (SSFP) cine images were acquired during expiratory breath-holds and with retrospective electrocardiographic gating. Imaging parameters were: slice thickness 5 mm; no gap; repetition time 2.9 ms; echo time 1.2 ms; flip angle 80°; field of view 340 to 380 mm^2^; matrix 256 × 146; bandwidth 930 Hz/px; 30 phases per R-R-interval. In a subgroup of 13 patients, prosthetic orifice was additionally depicted using fast gradient echo (FGRE) cine loops. Imaging parameters were: slice thickness 5 mm, no gap, repetition time 6.5 ms, echo time 3.1 ms; flip angle 15°; field of view 340 to 380 mm^2^, matrix 192 × 125, bandwidth 260 Hz/px, 25 phases per R-R interval.

**Figure 1:**
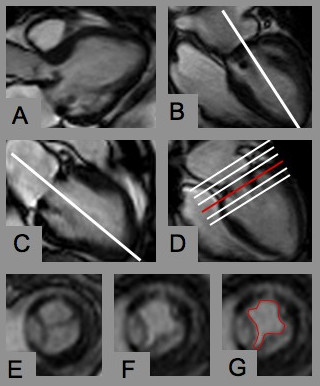
**Slice positioning for prosthetic orifice planimetry**. A) and B) 3- and 4-chamber-view showing the mitral bioprosthesis. Planning the next slice in the 4-chamber view along the transprosthetic jet *(white line)*. C) Again planning the next plane centrally through the transprosthetic jet. D) Positioning a stack of slices perpendicular to the jet covering the prosthesis during cardiac motion. E) and F) Cross section of the mitral bioprosthesis in systole and diastole. G) Manual contouring of the largest diastolic orifice area.

Positioning of the cine loops for orifice planimetry was performed in a stepwise fashion to ensure accurate positioning of the imaging plane perpendicular to the transprosthetic jet (Figure 1): Based on the four- or three-chamber-view, two subsequent planes were positioned centrally through the transprosthetic jet. Then, a stack of about six slices perpendicular to the jet was planned. Planimetry of the diastolic orifice area was performed in a single frame based on cross-references using the software CMR^42 ^(CIRCLE Cardiovascular Imaging, Calgary, Alberta, Canada).

Images were rated regarding overall image quality on a scale from 0 to 3 (0 poor, non-diagnostic; 1 impaired image quality that may lead to misdiagnosis; 2 moderate; 3 good). To test intra-observer variability of CMR planimetry, one investigator (FvK) analyzed all examinations twice with a latency of at least three months. To test inter-observer variability, two independent observers (FvK; AR), who were unaware of each other's interpretations, performed planimetry.

Examination of ventricular function was performed by acquisition of SSFP cine images in standard four- and two-chamber views, and where wall motion abnormalities were present, in a stack of short axes covering the whole left ventricle (imaging parameters as described elsewhere [3]).

### Statistical Analysis

Results are presented as mean ± standard deviation. Correlations between orifice areas obtained by TTE and CMR were analyzed using the Spearman rank order correlation test and are displayed as scattergrams with regression line. Bland-Altman analysis was used to evaluate the agreement between orifice area measurements according to the various methods, and to determine observer-related variability. Statistical significance was implied where p < 0.05. Statistical analyses were performed using Prism 5.0 (Graphpad Software, La Jolla, CA, USA).

## Results

### Echocardiographic assessment of mitral bioprostheses

Peak and mean transprosthetic pressure gradients were 11.5 ± 4.9 mmHg (range 6-22 mmHg) and 4.6 ± 2.5 mmHg (range 2-11 mmHg). Mean pressure half time was 110.8 ± 26.9 ms (range 85-192 ms). Mean prosthetic orifice area was 2.1 ± 0.3 cm^2 ^(range 1.5-2.6 cm^2^). Prosthetic regurgitation was present in 16 subjects (15 mild, 1 severe). Aortic and tricuspid regurgitation were present in 16 (13 mild, 3 moderate) and 18 (10 mild, 8 moderate) subjects. There was no patient without any left-sided valvular regurgitation. Five subjects had concomitant biological aortic valve replacement.

### Feasibility of CMR to image the mitral bioprosthesis

**Figure 2:**
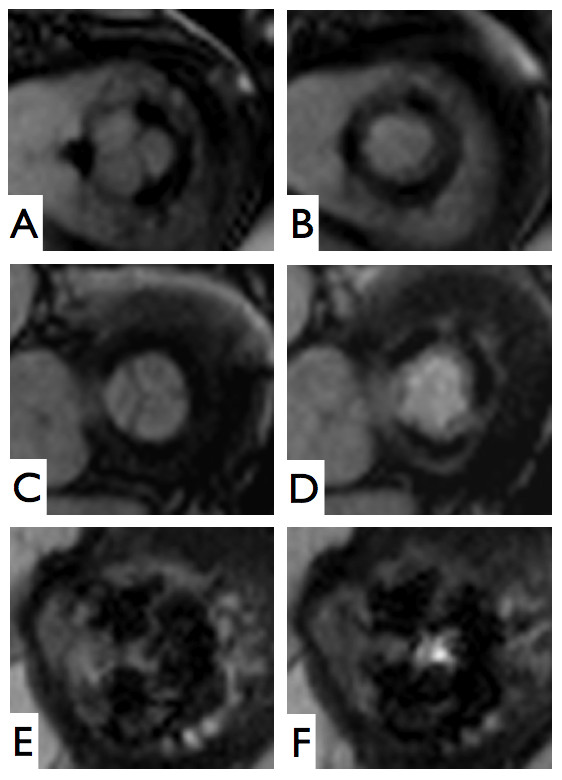
**Mitral bioprostheses imaged by CMR**. A) and B) Hancock^® ^#29, implanted 1998, imaged during sinus rhythm. C) and D) Labcore^® ^#29, implanted 2005, imaged during atrial fibrillation. E) and F) Perimount^® ^#31, implanted 2001, imaged during atrial fibrillation, with strong artifacts caused by the stent.

The image quality was rated as "good" in 10 (Figure 2, Additional file 1), "moderate" in 6 and "impaired that may lead to misdiagnosis" in 2 subjects. The reason for the significantly impaired image quality in 2 patients were considerable artifacts caused by the prosthetic stent in one subject (Figure 2, Additional file 2), and artifacts caused by mal-triggering in atrial fibrillation and insufficient breath holding in the other. The image quality rating differed significantly between evaluations in the presence and absence of arrhythmias (2.2 vs. 3.0; p = 0.013). All subjects in sinus rhythm had "good" image quality, and all subjects with "moderate" or "impaired" image quality had arrhythmias. On the other hand, even 4 subjects with atrial fibrillation achieved "good" image quality. In five subjects, an impaired opening movement of at least one valvular cusp was detected by CMR (Additional file 3).

### Accuracy of quantification of the mitral prosthetic orifice area by CMR

**Figure 3:**
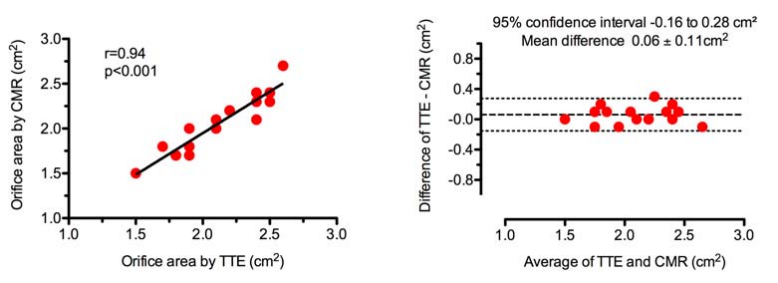
**Comparison of CMR and TTE**. Scattergram *(left) *and Bland-Altman plot *(right) *comparing the prosthetic orifice areas obtained by CMR and TTE. *(Middle dotted line indicates the mean difference; outer dotted lines indicate the limits of 2 standard deviations of the difference)*.

Mean prosthetic orifice area obtained by CMR was 2.1 ± 0.3 cm^2 ^(n = 18; range 1.5 to 2.7 cm^2^). Correlation analysis with TTE was significant (r = 0.94; p < 0.001) (Figure 3). Bland-Altman analysis revealed a 95% confidence interval ranging from -0.16 to 0.28 cm^2 ^when comparing TTE- and CMR-derived orifice areas (mean difference 0.06 ± 0.11 cm^2^, range -0.1 to 0.3 cm^2^) (Figure 3).

Bland-Altman analysis of TTE- and CMR-derived orifice areas in dependence of the heart rhythm revealed a 95% confidence interval from -0.27 to 0.20 cm^2 ^(mean difference -0.03 ± 0.12 cm^2^, range -0.2 to 0.1 cm^2^) in subjects with sinus rhythm (n = 6), and from -0.28 to 0.13 cm^2 ^(mean difference -0.08 ± 0.11 cm^2^, range -0.3 to 0.1 cm^2^) in subjects with arrhythmias (n = 12). Bland-Altman analysis of TTE- and CMR-derived orifice areas referring to image quality showed a 95% confidence interval from -0.20 cm^2 ^to 0.18 cm^2 ^(mean difference -0.01 ± 0.10 cm^2^, range -0.2 to 0.1 cm^2^) in "good" image quality (n = 10), and from -0.30 to 0.05 cm^2 ^(mean difference -0.13 ± 0.09 cm^2^; range -0.3 to 0.0 cm^2^) in "moderate" and "impaired" image quality (n = 8).

Agreement between both methods was similar for orifice areas smaller than the median 2.1 cm^2 ^(n = 8; 95% confidence interval -0.17 to 0.27 cm^2^; mean difference 0.05 ± 0.11 cm^2^) and ≥2.1 cm^2 ^(n = 10; 95% confidence interval -0.17 to 0.31 cm^2^; mean difference 0.07 ± 0.12 cm^2^).

**Figure 4:**
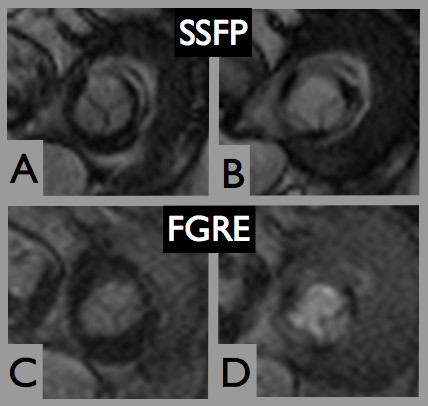
**Comparison of SSFP and FGRE**. Hancock^® ^#31, implanted 2008, imaged during sinus rhythm by SSFP (A and B) and FGRE (C and D).

Regarding the application of FGRE for planimetry, Bland-Altman analysis revealed a 95% confidence interval ranging from -0.37 to 0.31 cm^2 ^when comparing SSFP and FGRE (mean difference -0.03 ± 0.17 cm^2^; range -0.3 to 0.3 cm^2^) and from -0.29 to 0.35 cm^2 ^when comparing FGRE and TTE (mean difference 0.03 ± 0.16 cm^2^; range -0.2 to 0.4 cm^2^). Figure 4 shows a mitral bioprosthesis imaged by FGRE and SSFP (Additional file 4).

### Intra- and inter-observer variability of CMR planimetry

Repeated measurements of prosthetic orifice area by CMR-planimetry by one investigator resulted in an intra-observer variability of 4.5 ± 2.9% (range 0 to 9.5%). Bland-Altman analysis revealed a mean difference of 0.03 ± 0.11 cm^2 ^(range -0.2 to 0.2 cm^2^; 95% confidence interval -0.19 to 0.25 cm^2^). Assessment by two blinded observers revealed an inter-observer variability of 7.9 ± 5.2% (0.0 to 21.1%). Bland-Altman analysis revealed a mean difference of -0.08 ± 0.19 cm^2 ^(range -0.4 to 0.2 cm^2^; 95% confidence interval -0.30 to 0.46 cm^2^).

## Discussion

Planimetry is the most direct, and thus should be the most stable approach to determine a valvular orifice area. It is not derived from other hemodynamic and geometric calculations and hence is widely independent from hemodynamic conditions. For mitral valves, planimetry showed the best correlation with anatomical mitral valve area as assessed on explanted valves [9] and is therefore considered as the reference measurement of mitral valve area [10]. Based on the successful application of CMR planimetry in aortic bioprostheses, we hypothesized that CMR planimetry of mitral bioprostheses would also provide accurate orifice areas, as long as the image quality allows for a clear-cut delineation of the prosthetic cusp borders.

### Feasibility of CMR to image mitral bioprostheses

We have demonstrated the feasibility of CMR to image mitral bioprostheses, even in the presence of arrhythmias. Indeed, only 6 subjects presented in sinus rhythm, while 1 had frequent ventricular extrasystoles and 11 were experiencing atrial fibrillation. This incidence of arrhythmia is typical for patients previously suffering from mitral regurgitation or stenosis and having atrial dilatation [11]. As SSFP cine movies collect data over several cardiac cycles, the image quality negatively correlates with the extent of arrhythmia and with increasing heart rate. Even in the present sample, differences in image quality were observed between patients in sinus rhythm and those with arrhythmia. Nevertheless, none of the examinations hat to be excluded due to non-diagnostic image quality. But, optimal heart rate control is recommended preceding the assessment of a mitral bioprosthesis by CMR.

A strong stent artifact appeared in one subject with a Perimount^® ^prosthesis, whereas this phenomenon did not occur in the remaining subjects, who received other types of devices. This underlines that some prostheses are less suitable for CMR assessment and that the type of the valve makes an important difference concerning image quality. The differences probably depend on the shape and quantity of metal within the stent, and on its composition, which is a cobalt-chromium spring alloy in the Perimount^®^. Further trials including a greater variety of prosthetic types are warranted.

Artifacts caused by turbulent flow did not constitute a significant challenge in mitral prostheses, which can be attributed to the low transmitral blood flow velocity. Therefore, SSFP is the sequence of choice despite its general susceptibility for turbulent flow [12,13]. In rare cases with flow artifacts, less flow sensitive FGRE [13] may be applied. However, mitral prosthetic orifice areas obtained by FGRE showed less agreement with TTE than those determined by SSFP, which is in accordance with the use of FGRE to evaluate aortic stenosis [14].

### Accuracy of CMR to quantify the orifice area of mitral bioprostheses

Direct comparison of the orifice areas obtained by CMR planimetry with a gold standard would be desirable to confirm our hypothesis that CMR of mitral bioprostheses provides accurate prosthetic orifice areas. However, a true gold standard does not exist. Even when evaluating native mitral stenosis it is recommended that calculated mitral valve area by any one method should not be used as the single measure of severity of stenosis [15]. Orifice areas provided by the manufacturers are not a suitable gold standard, as they mostly comprise the geometric orifice area in-vitro, which is different from the orifice area effectively used in-vivo [16]. Planimetry using echocardiography is complicated by difficulties in identifying the correct cross-section [17,18]. Three-dimensional imaging may improve echocardiography [19,20], but has not yet entered routine clinical practice. The accuracy and reproducibility of the continuity equation are hampered by the number of contributing measurements, which is even more problematic in arrhythmias [18]. Furthermore, this algorithm is inappropriate in those with aortic or mitral regurgitation, as in our study subjects. The validity of pressure half time derived orifice areas, as traditionally applied in native mitral stenosis [8], is unclear in prosthetic valves, because of the dependence on left ventricular and left atrial compliance, on left atrial pressure and heart rate [1,21-23]. Nevertheless, in the absence of a better standard, and bearing these limitations in mind, we selected this parameter as the most appropriate for our study.

CMR planimetry of mitral bioprosthetic orifice area was significantly correlated with data obtained by TTE. The agreement between both methods was similar to the successful application of CMR to assess native mitral valve stenosis by Djavidani et al. (*r *= 0.98; mean difference 0.03 ± 0.09 cm^2^) [24] and even superior compared to another series by this group (r = 0.81; mean difference 0.13 ± 0.24 cm^2^) [4]. Compared to the application of computed tomography to assess native mitral valves in comparison to TTE-planimetry (r = 0.88; mean difference 0.20 ± 0.17 cm^2^) [25], the present results in mitral bioprostheses showed even closer agreement. In comparison to our results of CMR and TTE to assess aortic bioprostheses (r = 0.82; mean difference -0.02 ± 0.24 cm^2^) [3], the limits of agreement of CMR and TTE in mitral prosthetic assessment were smaller.

The present results demonstrated similar agreement between CMR- and TTE- derived orifice areas both in the presence or absence of arrhythmias. There was a tendency towards underestimation of orifice areas using CMR compared to TTE in examinations rated with "moderate" and "impaired" image quality, which may be explained by less accurate delineation of the border between blood and prosthetic cusps. However, the sample size is too small to draw firm conclusions regarding the influence of arrhythmias and image quality on prosthetic orifice assessment; thus, larger series are required.

CMR and TTE showed agreement that was independent of prosthetic orifice area. However, the study sample only contained 3 patients with an orifice area ≤1.7 cm^2 ^and just 1 patient with an orifice area ≤1.5 cm^2^. Prosthetic dysfunction may be associated with increased turbulent flow causing significant flow artifacts, which may influence image interpretation. Therefore, larger clinical trials are required to extend the use of CMR to the assessment of dysfunctional mitral prostheses.

The inter- and intra-observer variabilities of CMR planimetry were low and within the range of accepted echocardiographic results that report 5% to 8% variability for experienced observers examining native aortic valves [26]. Observer dependency was also comparable to data obtained using CMR in aortic bioprostheses with an intra- and interobserver variablity of 6.7% and 11.5%, respectively [3].

### Clinical impact of CMR to quantify the orifice area of mitral bioprostheses

TTE will remain the first choice for the assessment of mitral bioprostheses due to its ready availability, even bedside in the intensive care, cost effectiveness, non-invasiveness and proven accuracy. Bioprosthetic mitral valves constitute a very small number of patients undergoing mitral valve surgery, and of those an even smaller percentage will have unfavorable acoustic windows making Doppler interrogation unreliable. Nevertheless the present results demonstrated that for selected subjects, e.g. in case of insufficient acoustic windows, discordant echocardiographic results or within clinical research regarding patient-prosthesis mismatch [27], CMR planimetry poses a reliable non-invasive method to quantify the prosthetic orifice area.

## Conclusions

We demonstrated the feasibility of CMR to image mitral bioprostheses and to accurately assess the orifice area with close agreement to echocardiography and low observer dependency. Even though image quality decreased with the presence of arrhythmias and seemed to depend on the prosthetic type, all CMR examinations provided diagnostic image quality. Further clinical studies including patients with prosthetic dysfunction and a greater variety of prosthetic types are necessary to fully elucidate the applicability of these findings to a wider population.

## Competing interests

The authors declare that they have no competing interests.

## Authors' contributions

FKB, AR, RW and JSM have been involved in conception and design. FKB recruited the subjects. FKB, AR, RW, JSM have been involved in data acquisition. FKB, AR, RW and JSM analyzed the images. FKB and JSM analyzed and interpreted the data. FKB performed the statistical analysis. JSM supervised the study. FKB drafted the manuscript. JSM critically revised the manuscript and has given final approval of the version to be published. All authors had full access to the data and take responsibility for its integrity. All authors have read and approved the final manuscript.

## Supplementary Material

Additional file 1 **Mitral bioprosthesis imaged by SSFP**. SSFP cine movie of a mitral bioprosthesis representing good image quality (Labcore^® ^#27, implanted 2005, in sinus rhythm). Click here for file

Additional file 2 **Stent artifact**. SSFP cine movie of a mitral bioprosthesis with markedly impaired image quality due to artifacts caused by stent material (Perimount^® ^#31, implanted 2001, in atrial fibrillation). Click here for file

Additional file 3 **Prosthetic dysfunction**. SSFP cine movie of a mitral bioprosthesis with structural deterioration leading to incomplete diastolic opening of the valvular cusps (Hancock^® ^#33, implanted 2000, in atrial fibrillation). Click here for file

Additional file 4 **Mitral bioprosthesis imaged by FGRE**. FGRE cine movie of a mitral bioprosthesis (Hancock^® ^#31, implanted 2008, in sinus rhythm). Click here for file
